# Endophytic fungi in buckwheat seeds: exploring links with flavonoid accumulation

**DOI:** 10.3389/fmicb.2024.1353763

**Published:** 2024-02-20

**Authors:** Lingyun Zhong, Bei Niu, Dabing Xiang, Qi Wu, Lianxin Peng, Liang Zou, Jianglin Zhao

**Affiliations:** ^1^College of Preclinical Medicine, Chengdu University, Chengdu, China; ^2^Key Laboratory of Coarse Cereal Processing, Ministry of Agriculture and Rural Affairs, Chengdu, China

**Keywords:** *Fagopyrum*, seed, high-throughput sequencing, endophytic fungi, flavonoids

## Abstract

Buckwheat is a famous edible and medicinal coarse cereal which contain abundant of bioactive flavonoids, such as rutin. In this study, the composition and diversity of endophytic fungi in eight different buckwheat seeds were analyzed by high-throughput sequencing of ITS rDNA. Results showed that, the fungal sequences reads were allocated to 272 OTUs, of them, 49 OTUs were shared in eight buckwheat seeds. These endophytic fungi could be classified into 6 phyla, 19 classes, 41 orders, 79 families, 119 genera, and 191 species. At genus level, *Alternaria* sp. was the domain fungal endophyte. Besides, fungal endophytes belonged to the genera of *Epicocum, Cladosporium, Botrytis, Filbobasidium, Stemphylium*, and *Vishniacozyma* were highly abundant in buckwheat seeds. The total flavonoids and rutin contents in tartary buckwheat cultivars (CQ, XQ, CH, K2) were much higher than those in common buckwheat cultivars (HT, T2, T4, T8). For tartary buckwheat cultivars, the total flavonoids and rutin contents were ranging from 2.6% to 3.3% and 0.9% to 1.3%, respectively. Accordingly, the tartary buckwheat samples displayed stronger antioxidant activity than the common buckwheat. Spearman correlation heat map analysis was successfully found that certain fungal species from the genera of *Alternaria, Botryosphaeria, Colletorichum* and *Diymella* exhibited significant positive correlation with flavonoids contents. Results of this study preliminary revealed the fungi-plant interaction relationship at secondary metabolite level, and could provide novel strategy for increasing the flavonoids accumulation of buckwheat seeds, as well as improving their quality.

## Introduction

Plant endophytic fungi usually reside in healthy tissues and organs without causing disease symptoms in host plants. These microorganisms are widely distributed in plant roots, stems, leaves, flowers, fruits, or seeds, but only a small part of endophytic fungi could be cultured and isolated by surface disinfection from plants (Zhong et al., 2017; Pozo et al., 2021). During the process of long-term co-evolution, the endophytic fungi have developed a strong symbiotic relationship with their host plants. Many fungal endophytes can help host plants resist adverse stresses (biotic or abiotic) by bringing beneficial effects on plant photosynthesis, plant hormones, secondary metabolites, the antioxidant defense system, or carbon and nitrogen metabolism (Chen et al., 2021; Mirsam et al., 2021; Devi et al., 2023; Omomowo et al., 2023). Furthermore, the endophytic fungi can benefit their host plants by synthesizing a variety of bioactive enzymes or compounds. Endophytic fungi themselves can also act as effective elicitors, which may increase the contents of pharmacologically active substances synthesized by host plants (Khan et al., 2015; Zhao et al., 2015; Gao et al., 2023). Moreover, the fungal endophytes could promote host plant growth or metabolite accumulation by inducing the upregulation of related gene expression (Chen et al., 2023).

Seeds, as an important reproductive organ of plants, are parasitized by various endophytic fungal strains (Chen et al., 2018; Yan et al., 2022). These fungal endophytes may invade and reside in the seed coat, germ layers, or/and endosperm (Barret et al., 2015; Verma et al., 2019). Comparing to foliar endophytes, certain seed fungal endophytes strains have shown special biological characteristics, such as forming endospores, producing phytase, or moving between cells, which could help endophytic fungi invade their host seeds or maintain vitality in harsh conditions (Chen et al., 2021; Geisen et al., 2021; Murawska-Wlodarczyk et al., 2022). Especially some seed endophytic fungi can transfer from generation to generation through seed dispersal (seed-borne). Their presence in seedlings through vertical dispersal may provide beneficial symbionts for the next generation (Shearin et al., 2018). In recent years, numerous fungal endophytes associated with plant seeds have been identified from rice, corn, quinoa, barley, wheat, soybean, grapes, zucchini, dendrobium, plantain, Yunnan quince, spruce, or other host plants (Almeida dos Reis et al., 2022; Necajeva et al., 2023; Sun et al., 2023). The most abundant fungi identified from plant seeds belong to Ascomycetes and Basidiomycetes, mainly including the genera of *Penicillium*, *Mucor*, *Rhizoctonia*, *Talaromyces*, *Aspergillus*, *Fusarium*, *Phomopsis*, *Colletotrichum*, *Paecilomyces*, *Alternaria*, *Trichoderma*, and *Cercospora* (Kovacec et al., 2016; Shahzad et al., 2018; Patil et al., 2021).

Buckwheat (*Fagopyrum* sp.) is a dicotyledons plant belonging to the family Polygonaceae (Tang et al., 2019). By now, there are more than 20 species of buckwheat plants around the world, and it is widely cultivated in China, Russia, Ukraine, Poland, the United States, and Brazil (Fan et al., 2019). As a famous edible and medicinal coarse cereal, buckwheat is rich in protein, fat, dietary fiber, vitamins, and other valuable nutrients (Dziadek et al., 2018; Zou et al., 2021). Notably, buckwheat seeds contain an abundance of bioactive flavonoids that show multiple pharmacological activities, such as antibacterial, antioxidant, hypoglycemic, anti-tumor, anti-inflammatory, analgesic, and liver protective effects (Peng et al., 2015; Ninomiya et al., 2022). Particularly, the buckwheat seeds are rich in rutin, which is considered among the top therapeutically active phytochemicals (Semwal et al., 2021). As the major bioactive flavonoid of buckwheat, the antioxidant properties of rutin have been well documented and demonstrated a wide range of pharmacological applications. Up to now, buckwheat is the only known cereal that contains rutin and has been used as a raw material for rutin-rich food products (Suzuki et al., 2023). Buckwheat and its relevant products, such as buckwheat rice, buckwheat noodles, buckwheat tea, buckwheat biscuits, and buckwheat sprouts, are increasingly favored by consumers. Buckwheat grains and their related products are widely consumed around the world. They are promising raw materials for producing healthy coarse cereal products, and there is a huge demand for high-quality buckwheat seeds rich in flavonoids. It would be interesting and meaningful to elucidate the fungal compositions and diversities of buckwheat seeds. As it is an effective strategy for improving the flavonoid contents in the buckwheat seeds by using endophytic fungi elicitors, the possible relationships between the flavonoid accumulation and their endophytic fungi species were tried to be explored in this research.

## Materials and methods

### Plant materials

**Figure 1:**
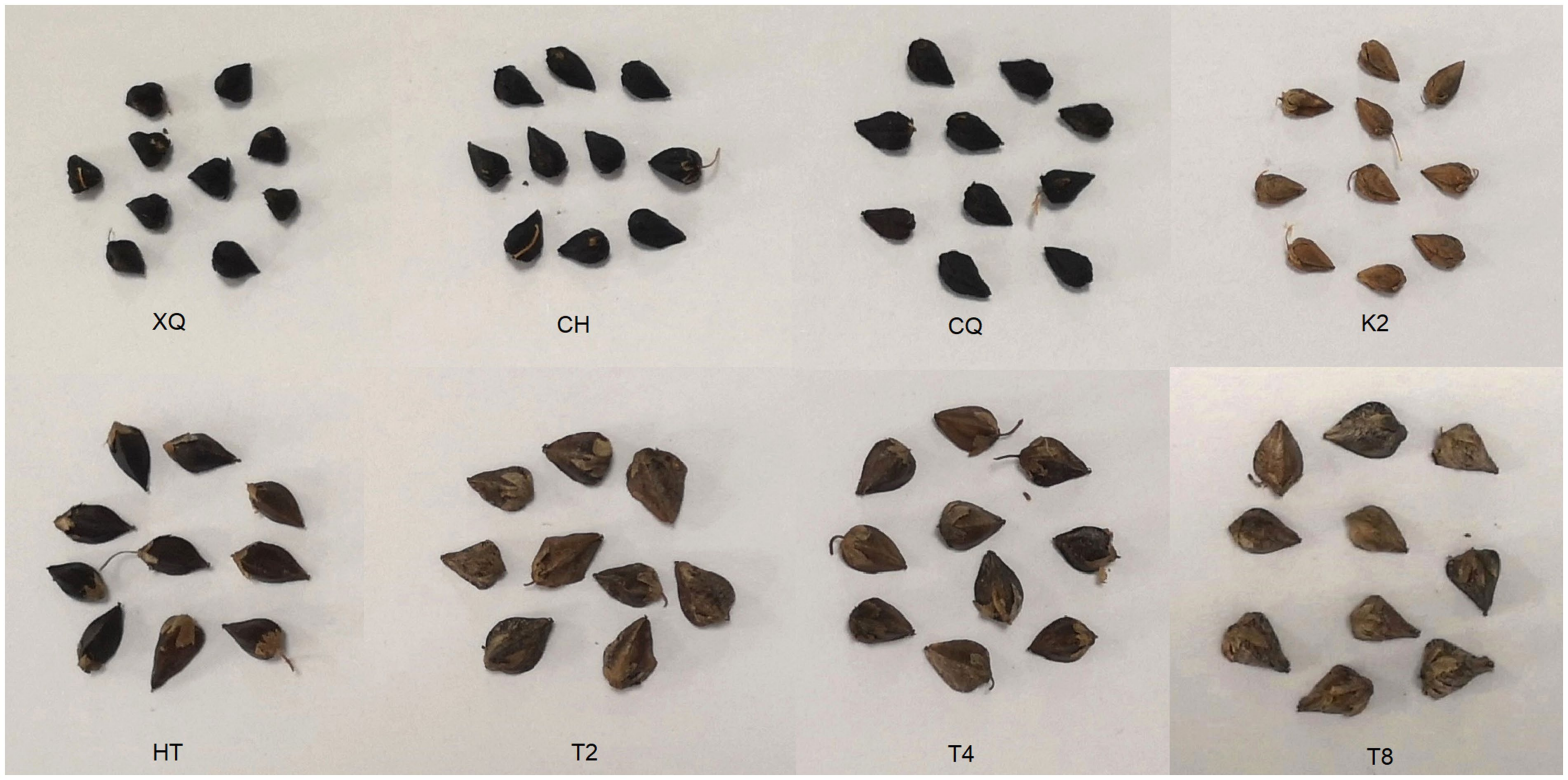
Tartary buckwheat (XQ, CH, CQ, and K2) and common buckwheat (HT, T2, T4, and T8) seed samples.

The tartary buckwheat (*Fagopyrum tataricum*) and common buckwheat (*F. esculentum*) seeds were collected from the Chengdu Jintang buckwheat breeding base (104°52′ E, 30°62′ N), Sichuan Province, China, in August 2020. Eight buckwheat cultivars were selected, including four tartary buckwheat cultivars: Chuanqiao (CH), Xiqiao (XQ), Kuciqiao (CQ), and Yunqiao (K2), and four common buckwheat cultivars: Honghua (HT), Shanxi (T2), Ningqiao (T4), and Ningtian (T8) (shown in Figure 1). All these seed samples were stored at −20°C, and sample voucher specimens were deposited at the Coarse Cereal Research & Development Center of Chengdu University.

### Samples preparation, DNA extraction, and Illumina MiSeq sequencing

#### Surface disinfection

Initially, buckwheat seeds were washed in running tap water, then surface sterilized in 75% ethanol for 30 s, a 2.5% sodium hypochlorite solution for 1 min, and then rinsed in micro-free distilled water three times (1 min for each time). The final washing water was inoculated into the nutrition medium to confirm the sterilization efficiency.

#### DNA extraction and PCR amplification

The genomic DNA of the seed endophytic fungi community was extracted using the Fast DNA^™^ Spin Kit for Soil (MP Biomedicals LLC, United States), and the metagenomic DNA extraction was conducted according to the user manual. Briefly, surface-sterilized buckwheat seeds were placed in a pre-cooled mortar and grinded into powder in liquid nitrogen. A lysing Matrix E tube was filled with 0.5 g of seed powder, 978 μL of sodium phosphate buffer, and 122 μL of MT buffer. The mixture was homogenized in a vortex mixer, then centrifuged at 14,000 *g for 8 min. The supernatant was transferred to a clean centrifuge tube, and 250 μL of PPS was added. To pellet precipitate, the sample was centrifuged at 14,000 *g for 5 min. For adjusting DNA-binding conditions, the supernatant was transferred to a new 15 mL tube with 1 mL of binding matrix solution, and the tube was inverted for 2 min. A measure of 500 μL of supernatant was discarded after being placed on a rack for 5 min. Then 600 μL of DNA solution was transferred to a SPIN filter tube to bind the DNA. The SPIN filter tube was centrifuged at 14,000 *g for 1 min and discarded the filtrate (repeat this step if necessary). To wash the SPIN filter, 500 μL of the prepared SEWS-M solution was added and centrifuged at 14,000 *g for 1 min. Then, it was centrifuged again at 14,000 *g for 2 min and air-dried in a SPIN filter for 5 min at room temperature. To elute the DNA, 80 μL of DES elution solution was added and centrifuged at 14,000 *g for 1 min. The metagenomic DNA solution in the catch tube was ready to use. DNA concentration and purity were checked by agarose gel electrophoresis and UV–vis spectrophotometer (Thermo Scientific, United States), respectively.

The ITS regions of the fungal 18S rDNA were amplified with primer pairs ITS1F (5′-CTTGGTCATTTAGAGGAAGTAA-3′) and ITS2R (5′-GCTGCGTTCTTCATCGATGC-3′) by an ABI GeneAmp^®^ 9700 PCR thermocycler (ABI, CA, United States). Amplification was performed in a 20-μL reaction mixture containing 10* PCR reaction buffer (2 μL), 2.5 mM dNTPs (2 μL), 10 ng genomic DNA, 5 μM primer (0.8 μL each), 5 U/μL of Taq DNA polymerase (0.2 μL), BSA (0.2 uL), and ddH_2_O (20 μL). The PCR program was as follows: pre-denaturation at 95°C for 3 min, followed by 27 cycles of denaturation at 95°C for 30 s, annealing at 55°C for 30 s, and extension at 72°C for 45 s, with a final extension at 72°C for 10 min. The PCR product was extracted from 2% agarose gel and purified using the AxyPrep DNA Gel Extraction Kit (Axygen Biosciences, United States), according to the manufacturer’s instructions, and quantified using a Quantus^™^ Fluorometer (Promega, United States).

#### High-throughput sequencing and statistical analysis

The purified PCR products were sequenced on an Illumina MiSeq PE300 platform/NovaSeq PE250 platform (Illumina, United States) according to the standard protocols by Majorbio Bio-Pharm Technology Co. Ltd. (Shanghai, China). The raw sequences were demultiplexed, quality-filtered by length and quality using fastp version 0.20.0, then merged with FLASH version 1.2.7. Operational taxonomic units (OTUs) with a 97% similarity cutoff were clustered using UPARSE version 7.1, and chimeric sequences were identified and removed. The taxonomy of each OTU representative sequence was analyzed by RDP Classifier version 2.2 against the UNITE ITS database. All the data were analyzed on the online platform of Majorbio Cloud Platform.^1^ The Chao, ACE, Shannon, Simpson, and Coverage indexes were used to estimate the fungal alpha diversity (Gihring et al., 2012). A Venn diagram and principal coordinate analysis (PCoA) were used to reveal microbial community diversity among different seed samples. The relative abundance of the fungal community was expressed by community barplots both on the phylum and genus levels. The correlations between the fungal communities (relative abundance before 50%) and flavonoid contents (or antioxidant abilities) were conducted by Spearman analysis and expressed by the Spearman correlation heatmap (Cui et al., 2022).

### Preparation of buckwheat flavonoid extracts

A portion of buckwheat seed powder (0.2 g) was immersed in the 75% ethanol (ratio of solid to liquid 1:40), and the flavonoid extract was obtained by ultrasonic extraction for 1 h. The prepared flavonoid extract was used for the detection of total flavonoid and rutin contents.

### Determination of flavonoid contents of buckwheat seed extracts

#### Determination of total flavonoid content

The total flavonoid content of the seed extracts was tested by the aluminum trichloride colorimetry according to our previous study with some modifications (Zhong et al., 2022). Generally, 0.5 mL of sample solution (seed extracts or rutin standard solution) was mixed with 2 mL of 10% aluminum chloride, 3 mL of 1 M potassium acetate, and 5.5 mL of 75% ethanol. Then the mixture was maintained for 30 min at room temperature, and the absorbance was read at 415 nm by a spectrophotometer. The total flavonoid content was expressed as milligrams of rutin equivalent (RE) per gram of extract.

#### Determination of rutin content

The rutin content of each buckwheat seed extract was determined by the high-performance liquid chromatography (HPLC) method, according to Zhao et al. (2014). The HPLC analysis was performed on a LC-20A system using a C_18_ column (4.6 mm × 150 mm, 5 μm, Diamonsil, Torrance, CA, United States) and an SPD-M10Avp diode-array detector (Shimadzu, Kyoto, Japan), which recorded at 350 nm. The mobile phase was A (methanol:water:acetic acid = 5:92.5:2.5) and B (methanol:water:acetic acid = 95:2.5:2.5). The gradient elution was as follows: 0–1 min, 20% B; 1–22 min, 20–36% B; 23–35 min, 36–60% B; 26–33 min, 60% B; 34–40 min, 60–20% B. The flow rate was set at 1.0 mL/min. The column temperature was set at 40°C, and the infection volume was 10 μL. The pure rutin compound (purity ≧ 98%) was used as the reference standard, and the quantification of the rutin content of the buckwheat extract was calculated using an external standard method.

### DPPH and ABTS radical scavenging activity assay

The DPPH radical scavenging capacity of buckwheat seed extracts was evaluated by the method described previously (Zhong et al., 2020). Briefly, 20 μL of buckwheat extract solution and 80 μL of DPPH solution (0.2 mg/mL) were added to a 96-well microplate and mixed. Then, the mixtures were incubated at 37°C for 0.5 h in the dark, and the absorbance was measured spectrophotometrically at 515 nm.

The ABTS radical scavenging activity was conducted according to the method of Sung and Lee (2010) with some modifications. A total of 200 μL of the ABTS radical solution and 15 μL of sample solution were mixed in the microplate for 1 min in the dark. The absorbance at 405 nm was immediately measured using a microplate spectrophotometer. The DPPH or ABTS radical scavenging activity was determined as

$\begin{array}{l} \mathrm{DPPH}/\mathrm{ABTS}\;\mathrm{radical}\;\mathrm{scavenging}\;\mathrm{ability}\;\left(\%\right)\\ =\left[\left(ODc-ODs\right)/ODc\right]\times 100\;\text{,} \end{array}$

where ODc = absorbance of the negative control (75% ethanol) and ODs = absorbance of the test sample. Butylated hydroxytoluene (BHT) was used as the positive control.

## Results

### Richness and diversity analysis of fungal communities

**Figure 2:**
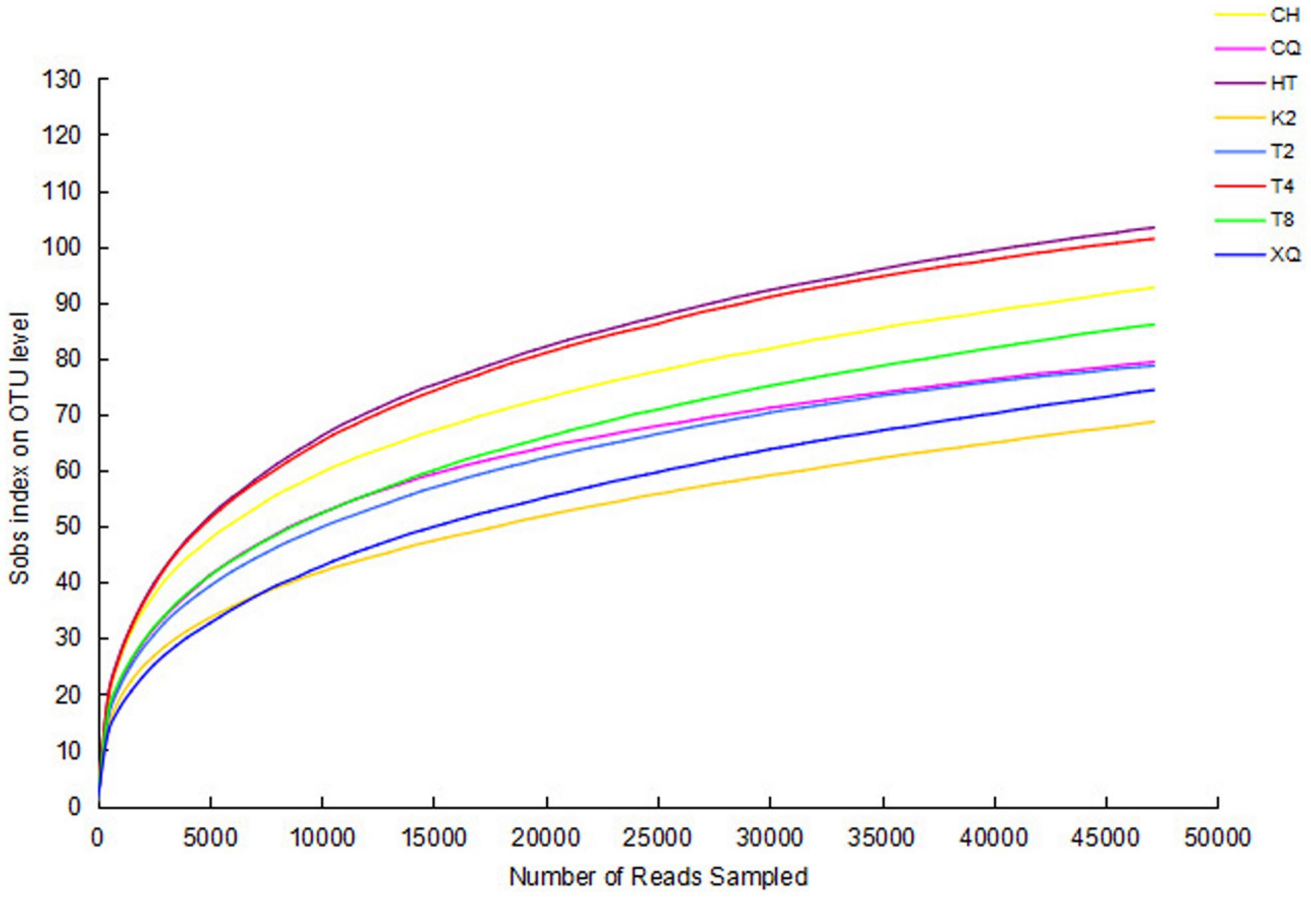
Rarefaction curves of all samples. The richness of endophytic fungi with different sequencing numbers is shown by the rarefaction curve. Sobs index was the number of OTUs actually observed. Higher Sobs index indicates a higher diversity of the fungal community. If the curves reach an asymptote, it indicates that more OTUs cannot be detected by increasing the sequencing data sequences.

The genomic sequences of endophytic fungi were obtained by high-throughput sequencing and analysis. The number of reads for the fungal sequences ranged from 59,158 to 71,497 (Table 1). To investigate the fungal community composition, the reads were classified into OTUs. Based on 97% sequence similarity, all these reads were allocated to 272 OTUs. The rarefaction curve was used to evaluate the richness of the fungal community and the sequencing depth of all these samples (Figure 2). As shown in Figure 2, all the rarefaction curves were flat and reached an asymptote, which indicated these sequences well represent the fungi diversity of eight buckwheat samples.

**Table 1:** ITS reads of endophytic fungi from *Fagopyrum* spp. seeds.

Sample	Reads	Base num	Mean length
CQ	63,441	15,107,948	238.10
XQ	63,626	15,191,976	238.73
CH	71,329	16,753,655	234.87
K2	60,275	14,336,329	237.91
HT	69,245	16,054,858	231.86
T2	71,497	16,915,150	236.58
T4	59,158	13,751,999	232.48
T8	63,317	14,830,918	234.24

### *α*-Diversity analysis of fungal communities

The alpha diversity index, including Shannon, Simpson, ACE, Chao, and Coverage, was used to reveal the diversity of seed endophytic fungal communities. As shown in Table 2, generally, the fungal communities from common buckwheat seeds (HT, T4, and T8) exhibited higher diversity than that of tartary buckwheat seeds (CQ, XQ, and K2), which was implied by a higher Shannon index and a lower Simpson index. T8 showed the highest diversity among all the seeds sampled; however, XQ displayed the lowest diversity. The richness of endophytic fungal communities was evaluated by the ACE and Chao indexes. CH revealed the highest fungal community abundance, followed by the HT.

**Table 2:** Fungal endophytes richness and diversity index of *Fagopyrum* spp. seeds.

Sample	Shannon	Simpson	ACE	Chao	Coverage
CQ	0.775	0.731	95.117	97.749	0.99960
XQ	0.617	0.768	124.661	116.404	0.99946
CH	1.219	0.502	139.321	127.829	0.99946
K2	0.716	0.733	142.951	117.528	0.99948
HT	1.416	0.401	136.160	129.442	0.99946
T2	0.973	0.622	92.529	89.804	0.99963
T4	1.592	0.334	121.771	119.016	0.99953
T8	1.549	0.343	115.302	116.769	0.99946

### Relative abundance of fungal communities

**Figure 3:**
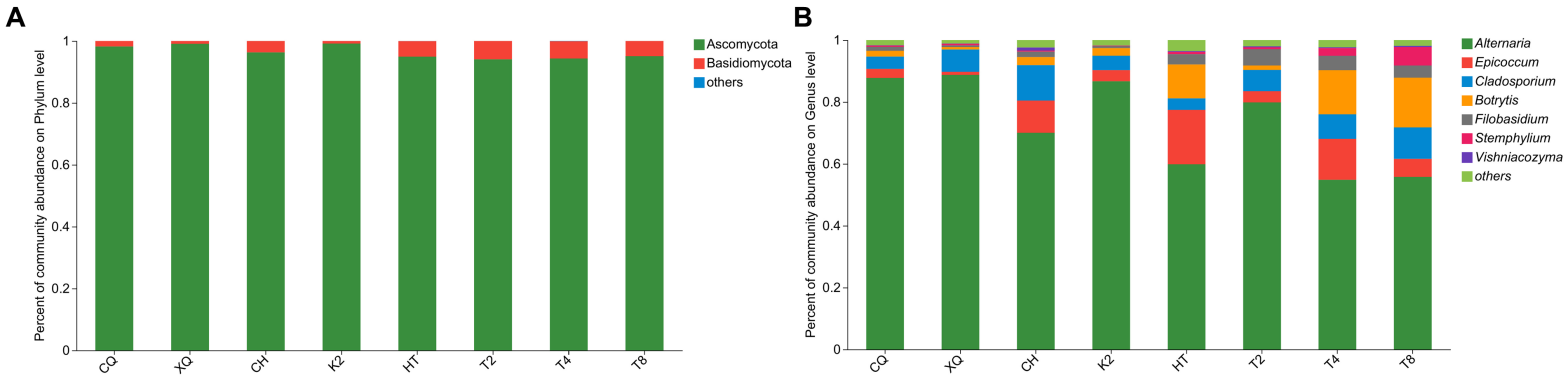
Relative abundance of fungal community at phylum **(A)** and genus **(B)** level.

According to the statistical analysis, a total of 272 OTUs were identified from all buckwheat seed samples. These OTUs could be generally classified into 6 phyla, 19 classes, 41 orders, 79 families, 119 genera, and 191 species. Based on 97% sequence similarity, the fungal community structure bars were constructed both at the phylum and genus level to analyze fungal compositions (shown in Figure 3). As shown in Figure 3A, most of the endophytic fungi were identified as Ascomycota, and Basidiomycota, and the rest of the endophytic fungi were grouped into the phyla of Chytridiomycota, Mortierellomycota, Rozellomycota and unclassified fungi, respectively. At the genus level, *Alternaria* sp. was the dominant fungal endophyte, and its community abundance rate was over 50% in all buckwheat seeds (Figure 3B). In addition, the fungal endophytes of *Epicocum*, *Cladosporium*, *Botrytis*, *Filobasidium*, *Stemphylium*, and *Vishniacozyma* were highly abundant in the buckwheat seeds. However, a large portion of OTU reads were still unclassified, and 109 reads were failed to be identified at the species level. These results revealed the fungal diversity in buckwheat seeds. Nevertheless, there were still plenty of unknown fungal endophytes that needed to be developed.

### Fungal composition comparisons and *ꞵ*-diversity analysis of the endophytic fungal communities

**Figure 4:**
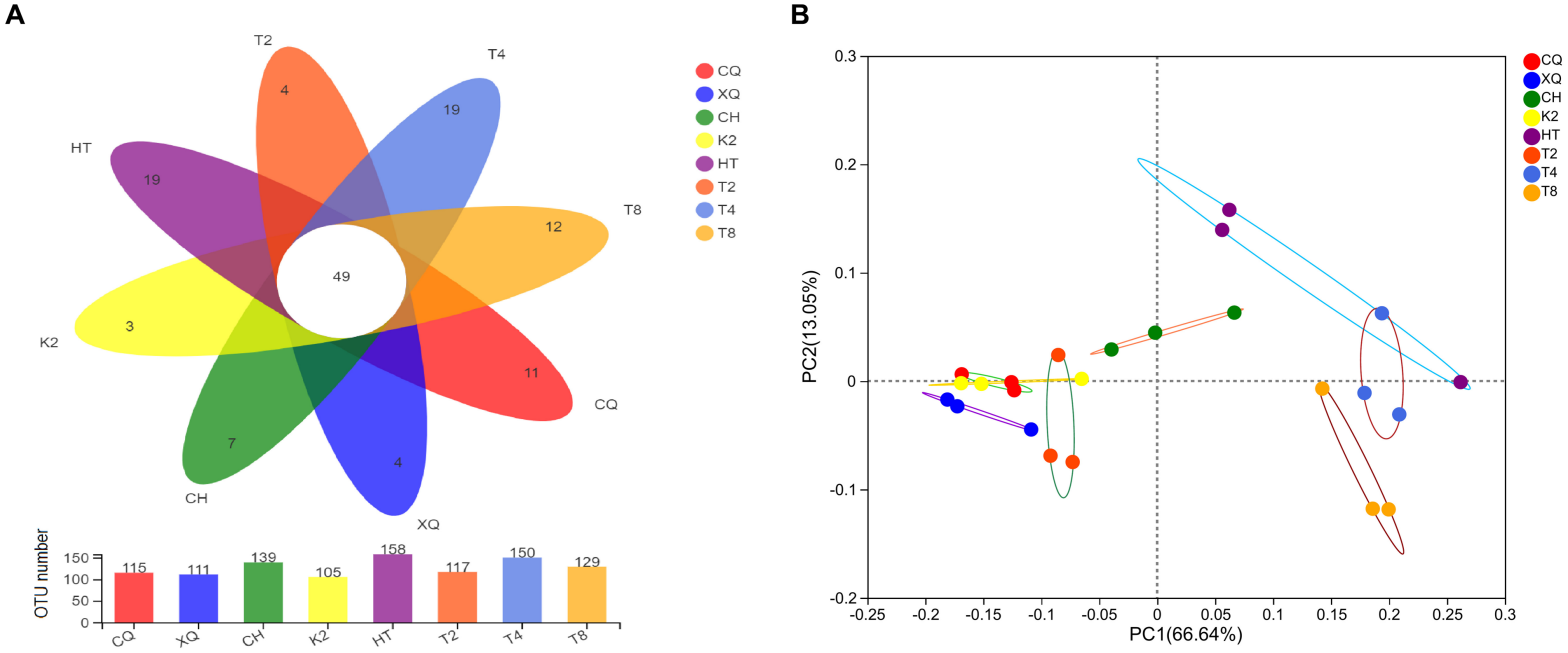
Venn diagrams **(A)** and PCoA analysis **(B)** of the buckwheat seed samples at OTU level. Different buckwheat seed samples are represented by different colors. Numbers in overlapping portions represent the number of species common in all buckwheat seeds **(A)** and dispersion of different points reveals the fungal diversity among buckwheat seeds **(B)**.

A Venn diagram was established to depict fungal community composition at OTU level among the seed samples of *Fagopyrum* spp. As shown in the Venn diagram (Figure 4A), the OTU numbers of the eight buckwheat seed samples ranged from 105 to 158. Of them, HT (common buckwheat) had the greatest OTU numbers, which represented the highest fungal diversity in all seed samples. Contrastingly, K2, which is one of the tartary buckwheat cultivars, had the lowest OTU numbers. From Figure 4A, we could find that 49 OTUs were shared in all 8 buckwheat samples. These results highlighted that fungal communities from different buckwheat seeds showed some similarity. These 49 common OTUs were identified in 20 genera, mainly *Alternaria*, *Botrytis*, *Cladosporium*, *Didymella*, *Filobasidium*, *Peyronellaea*, *Hannaella*, and *Vishniacozyma*. By analyzing seed unique OTUs, it could be found that common buckwheat HT and T4 both had the most unique OTU numbers of 19, followed by T8. Tartary buckwheat K2 only contained four unique OTUs, which was the lowest in these eight buckwheat seed samples.

*ꞵ*-diversity of the endophytic fungal communities was explored by PCoA analysis. As shown in Figure 4B, there was a significant separation of fungal communities among buckwheat samples. The PC1 and PC2 axes explained 66.75 and 13.08% of the variance in fungal species, respectively. The buckwheat samples CQ, XQ, K2, and T2 were clustered closely, which revealed the similarity among them. However, the fungal endophyte communities showed significant differences between HT and T8, which were revealed by distant clustering. These results proved that common buckwheat (T2, HT, T4, and T8) generally displayed higher fungal diversity than tartary buckwheat seeds (CQ, XQ, CH, and K2).

### Flavonoid contents and antioxidant activity of buckwheat seeds

The results of flavonoid contents and antioxidant activity are shown in Table 3. As it was shown, the total flavonoid and rutin contents were far higher in tartary buckwheat seeds (CQ, XQ, CH, and K2) than those in common buckwheat seeds (HT, T2, T4, and T8). In tartary buckwheat, the total flavonoid and rutin contents ranged from 2.6% to 3.3% and 0.9% to 1.3%, respectively. Of them, the K2 and XQ contained the highest total flavonoid contents, and the CQ had the lowest total flavonoid and rutin contents. For the common buckwheat, the total flavonoid contents ranged from 0.1 to 0.2%, and the rutin contents were all less than 0.1%. Accordingly, the tartary buckwheat seed extracts displayed stronger antioxidant activities than common buckwheat. For all the tartary buckwheat cultivars, the DPPH and ABTS free radical scavenging rates ranged from 77% to 80% and 80% to 89%, respectively. Different tartary buckwheat cultivars exhibited comparable DPPH or ABTS free radical scavenging abilities. However, for the common buckwheat cultivars, the DPPH and ABTS free radical scavenging rates were all less than 50%. The antioxidant activities of tartary buckwheat were much higher than those of common buckwheat.

**Table 3:** Flavonoid contents and antioxidant activities of the buckwheat seeds.

Sample	Total flavonoid content %	Rutin content %	DPPH free radical scavenging rate %	ABTS free radical scavenging rate %
CQ	2.680 ± 0.005^c^	0.970 ± 0.061^b^	77.910 ± 1.727^a^	87.254 ± 0.946^a^
XQ	3.128 ± 0.147^a^	1.287 ± 0.143^a^	79.426 ± 2.882^a^	88.606 ± 1.010^a^
CH	2.907 ± 0.138^b^	1.182 ± 0.209^a^	79.057 ± 0.986^a^	81.223 ± 2.900^b^
K2	3.291 ± 0.208^a^	1.197 ± 0.094^a^	78.402 ± 0.819^a^	88.146 ± 0.388^a^
HT	0.142 ± 0.028^d^	0.017 ± 0.003^c^	43.975 ± 2.308^bc^	36.983 ± 4.777^d^
T2	0.130 ± 0.042^d^	0.006 ± 0.002^c^	47.623 ± 2.938^b^	38.794 ± 1.959^cd^
T4	0.151 ± 0.027^d^	0.005 ± 0.003^c^	40.205 ± 4.393^c^	34.183 ± 1.437^d^
T8	0.186 ± 0.009^d^	0.020 ± 0.006^c^	45.246 ± 3.013^b^	42.471 ± 5.374^c^

Different letters (i.e., a–d) indicated significant differences among the treatments at *p* = 0.05.

### The correlations between endophytic fungal species, flavonoid contents, and antioxidant activities

**Figure 5:**
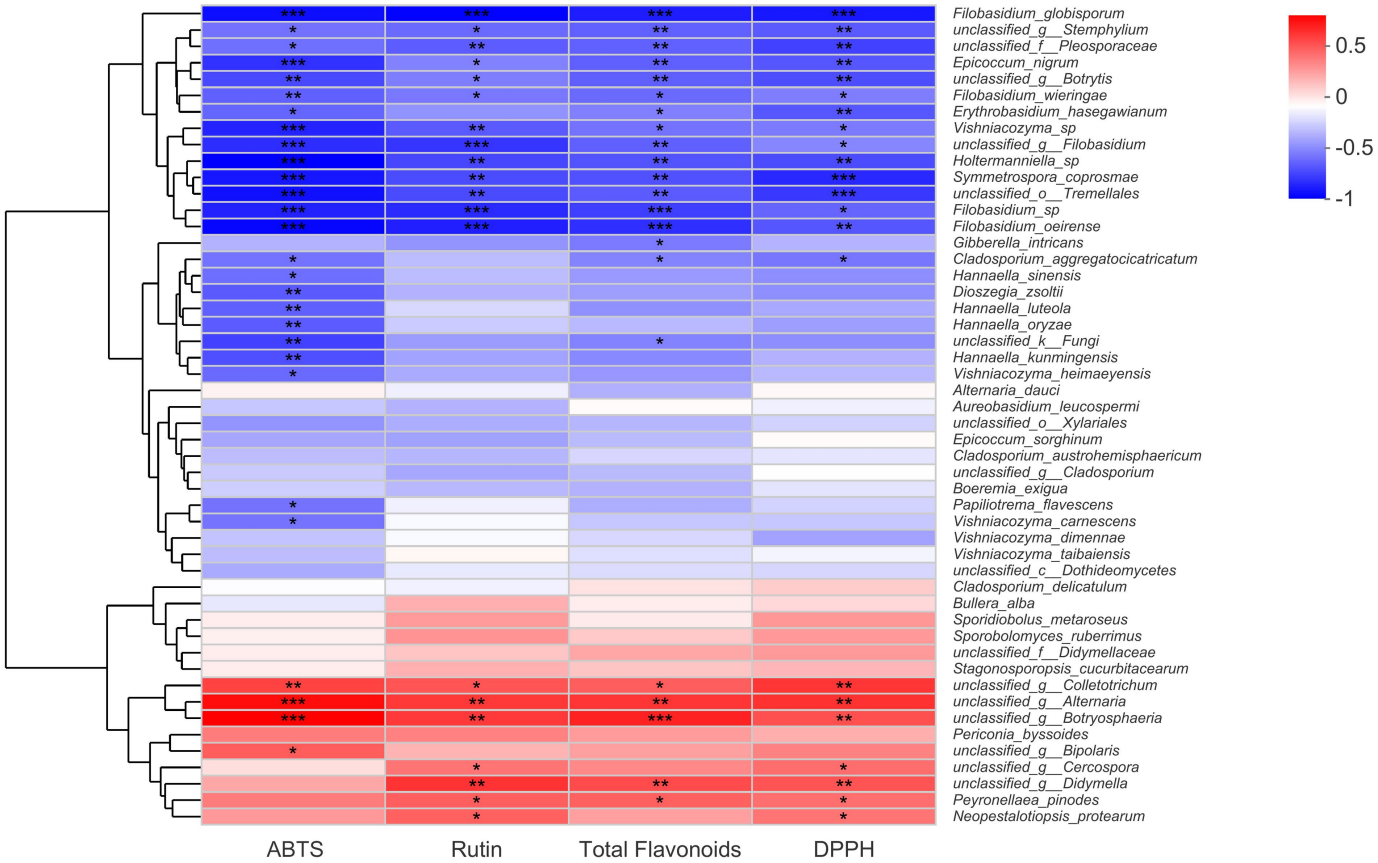
Spearman correlation analysis of fungal species, flavonoid content, and antioxidant activities of buckwheat seeds. * indicate the difference is significant at *p* < 0.05, ** indicate the difference is significant at *p* < 0.01; only the top 50% of fungi species were displayed.

A Spearman correlation heat map about fungal endophytes, flavonoid contents, and antioxidant activities was constructed in this part, and the results were presented in Figure 5. Evidently, from the heat map chart, we could find out that certain fungal species from the genera *Alternaria*, *Botryosphaeria*, *Colletotrichum*, and *Didymella* showed a significant positive correlation with total flavonoid content, rutin content, and antioxidant activity. However, it seemed there were more fungal species that displayed significant negative correlations with buckwheat flavonoids. Certain fungal species that belonged to the genera *Filobasidium*, *Stemphylium*, *Epicoccum*, *Symmetrospora*, *Vishniacozyma*, *Holtermanniella*, and *Botrytis* correlated negatively with total flavonoid content, rutin content, and antioxidant activities. Particularly, there were five *Filobasidium* strains that exhibited a negative relationship with flavonoid contents and antioxidant activities (Figure 5). It would be beneficial to further understand the physiological functions of *Filobasidium* sp. in buckwheat seeds. Nevertheless, there were still a large number of endophytic fungi that correlated with flavonoid accumulation and antioxidant activities. For these fungal endophytes, significant levels had not yet been reached, such as the fungal strains *Alternaria dauci*, *Aureobasidium leucospermi*, *Bullera alba*, *Boeremia exigua*, and *Epicoccum sorghinum*.

## Discussion

Buckwheat seeds/grains are abundant in flavonoid compounds and are famous for their notable antioxidant capacities. Flavonoids, such as rutin, quercetin, acacetin, cyanidin, myricetin, and kaempferol, are important antioxidant substances in buckwheat seeds, and these flavonoids cannot be found in the other grains of general cereal crops, such as rice or wheat (Almuhayawi et al., 2021; Kan et al., 2023). In this study, the total flavonoid and rutin contents were determined in eight different buckwheat cultivars. Results showed that the total flavonoid and rutin contents were much higher in tartary buckwheat seeds (CQ, XQ, CH, and K2) than those in common buckwheat seeds (HT, T2, T4, and T8). These results were consistent with previous reports by Xu et al. (2015) and Shao et al. (2011). Accordingly, the antioxidant activities of tartary buckwheat were much stronger than those of common buckwheat. The tartary buckwheat cultivars exhibited better medicinal potential than the common buckwheat.

Endophytic fungi are novel biological resources for exploitation in agriculture, medicine, and the food industry and present promising biotechnological potential, such as enzyme production, biocontrol agents, and plant- or growth-promoting agents (Zheng et al., 2016; Lindblom et al., 2018). In this study, we explored the endophytic fungi communities in different common buckwheat and tartary buckwheat seeds. Generally, common buckwheat displayed higher fungal diversity than tartary buckwheat seeds. By summarizing and analyzing the domain fungal endophytes of buckwheat seeds, *Alternaria* sp. was found to be dominant in all common buckwheat and tartary buckwheat seeds, and the genera *Botrytis*, *Epicocum*, *Cladosporium*, and *Filobasidium* were found to be highly abundant. However, there were a few differences in endophytic fungal communities of buckwheat seeds reported by other researchers. According to Bai et al. (2023), genera of *Sclerotinia*, *Cryptococcus*, and *Cladosporium* were found to be dominant in tartary buckwheat seeds, where seed fungal endophytes were sampled and identified by 18S rDNA amplicon sequencing after growing for 2 days in the incubator. In the study of Li et al. (2021), the genera *Cryptococcus*, *Aureobasidium*, *Botrytis*, *Acremonium*, and *Didymella* were found dominant in common buckwheat seeds. In the research of Mravlje et al. (2021), genera *Alternaria* and *Didymella* represented the vast majority of the fungal colonists in tartary buckwheat, and genera *Didymella* and *Epicocum* were two dominant fungi in common buckwheat. These results displayed differences in endophytic fungi compositions in buckwheat seeds, which might imply that the localities of buckwheat samples greatly affected the fungal compositions, and also that the sampling methods could affect the results of domain fungi in the seeds. Particularly, fungal endophytes belonging to the genus *Alternaria* deserved attention. As it is known, *Alternaria* sp. is usually found as an endophyte in many plant seeds such as wheat, rice, beans, coriander, sesame, and basil (Rehman et al., 2011; Gilardi et al., 2013; Mangwende et al., 2018; Kim and Lee, 2021). Some strains of *Alternaria* spp. displayed a symbiotic nature with host seeds (Mauricio-Castillo et al., 2020), but some of these seed-borne *Alternaria* strains might cause seed rot and seedling infections (Links et al., 2014). In buckwheat, *Alternaria* sp. might cause leaf spots (Qi et al., 2020). Buckwheat disease caused by *Alternaria* sp. should be given more attention, and prevention measures should be taken.

Further, in order to increase the flavonoid contents of buckwheat seeds and improve their quality, we tried to explore the possible relationships between fungal endophyte communities and flavonoid accumulation. It was found that, based on the Spearman correlation analyses, a portion of fungal species display a significant positive correlation with flavonoids, such as species from the genera *Alternaria*, *Botryosphaeria*, and *Didymella*. However, compared to fungi that exhibit positive correlations, there are more fungi that are negatively correlated with flavonoid components, such as those from *Filobasidium*, *Epicoccum*, and *Botrytis*. These results revealed endophytic fungi from buckwheat seeds interacted with their host plant at secondary metabolite levels, and the underlying mechanism would be worth studying further. We could infer from the results that fungal endophyte communities correlated positively with the flavonoid contents of buckwheat seeds, which might probably stimulate the host plant to synthesize more flavonoids and increase the flavonoid contents. Our previous research provided evidence to support this inference. In a previous study, it was found that the crude polysaccharide of the tartary buckwheat endophytic fungus *Alternaria* sp. could effectively increase the rutin and total flavonoid contents of buckwheat sprouts by activating the PAL activity (Zhao et al., 2014). As it is known, the endophytic fungi could produce metabolites similar to (or the same as) the host plant, and flavonoids, such as kaempferol and quercetin were successfully produced by the fungal endophytes (Alam et al., 2021). It could also be inferred that buckwheat endophytes might synthesize flavonoid components themselves, and the infection of endophytic fungi led to an increase in the flavonoids of their host plant. Even so, there is a possibility that both of the two mechanisms above existed during the process of infection by fungal endophytes. If the endophytes had a negative relationship with flavonoids, they might probably inhibit flavonoids synthesized through downregulating related genes. The answer to these questions would help better understand the interaction mechanism between endophytic fungi and hosts. This study laid the foundation for utilizing endophytic fungi to improve the flavonoid content and quality of buckwheat and also provided clues for the isolation and purification of buckwheat seed endophytic fungi in the next step. Moreover, understanding whether endophytic fungi in buckwheat seeds might pose potential pathogenic risks to host plants will contribute to the healthy and rapid development of the buckwheat industry.

## Conclusion

In this study, a culture-independent method was used to explore the endophytic fungi diversity of common and tartary buckwheat seeds. The ITS region of the fungal endophyte was sequenced by high-throughput sequencing. Fungal endophyte communities from common buckwheat and tartary buckwheat seeds showed certain similarity, and *Alternaria* sp. was the most dominant fungus in all seed samples. Generally, the common buckwheat displayed higher fungal diversity than that of the tartary buckwheat seeds. Specific fungal strains from the genera *Alternaria*, *Botryosphaeria*, and *Didymella* showed significant positive correlations with flavonoid contents, which could provide a promising strategy to increase flavonoid accumulation and improve the quality of buckwheat seeds.

## Data availability statement

The datasets presented in this study can be found in online repositories. The names of the repository/repositories and accession number(s) can be found at: https://www.ncbi.nlm.nih.gov/genbank/, PRJNA1042904.

## Author contributions

LZh: Data curation, Formal analysis, Funding acquisition, Methodology, Writing – original draft. BN: Project administration, Resources, Writing – original draft. DX: Methodology, Resources, Writing – review & editing. QW: Investigation, Methodology, Validation, Writing – original draft. LP: Supervision, Writing – review & editing. LZo: Supervision, Writing – review & editing. JZ: Formal analysis, Funding acquisition, Validation, Writing – original draft, Writing – review & editing.

## Footnotes

^1^ http://www.majorbio.com
